# Catchment vegetation and temperature mediating trophic interactions and production in plankton communities

**DOI:** 10.1371/journal.pone.0174904

**Published:** 2017-04-17

**Authors:** Anders G. Finstad, Erlend B. Nilsen, Ditte K. Hendrichsen, Niels Martin Schmidt

**Affiliations:** 1 Centre for Biodiversity Dynamics, Department of Natural History, NTNU University Museum, Norwegian University of Science and Technology, Trondheim, Norway; 2 Norwegian Institute for Nature Research, Trondheim, Norway; 3 Arctic Research Centre, Department of Bioscience, Aarhus University, Roskilde, Denmark; Uppsala Universitet, SWEDEN

## Abstract

Climatic factors influence the interactions among trophic levels in an ecosystem in multiple ways. However, whereas most studies focus on single factors in isolation, mainly due to interrelation and correlation among drivers complicating interpretation and analyses, there are still only few studies on how multiple ecosystems respond to climate related factors at the same time. Here, we use a hierarchical Bayesian model with a bioenergetic predator-prey framework to study how different climatic factors affect trophic interactions and production in small Arctic lakes. Natural variation in temperature and catchment land-cover was used as a natural experiment to exemplify how interactions between and production of primary producers (phytoplankton) and grazers (zooplankton) are driven by direct (temperature) and indirect (catchment vegetation) factors, as well as the presence or absence of apex predators (fish). The results show that increased vegetation cover increased phytoplankton growth rate by mediating lake nutrient concentration. At the same time, increased temperature also increased grazing rates by zooplankton. Presence of fish increased zooplankton mortality rates, thus reducing grazing. The Arctic is currently experiencing an increase in both temperature and shrub vegetation cover due to climate change, a trend, which is likely to continue. Our results point towards a possible future general weakening of zooplankton grazing on phytoplankton and greening of arctic lakes with increasing temperatures. At the same time, the impact of the presence of an apex predator indicate considerable local variation in the response. This makes direction and strength of global change impacts difficult to forecast.

## Introduction

Primary production and the trophic interactions between primary producers and consumers[1–3], are pivotal drivers on a wide array of ecosystem functions and services ranging from food production to carbon sequestration[4,5]. The interaction strength between trophic levels determine the ecosystem functions and services delivered, and changes in the relative strength between trophic levels will ultimately also change the management strategies needed to sustain resources for the future. One of the central tenets of contemporary ecological research is therefore to untangle effects of global warming on trophic interactions and ultimately on ecosystem functioning[6–10]. However, this inference is complicated by the multitude of interrelated and often correlated drivers. For example, it is becoming increasingly clear that climate alters ecosystem function through interactions between other global change drivers such as patterns of land use and through interactions across ecosystem boundaries[6,11–14]. To add to the complexity, organisms, trophic levels and ecosystem compartments are likely to be affected differently by different drivers.

The base of aquatic food webs are phytoplankton, constituting the largest photosynthesising biomass on earth[15], with zooplankton usually dominating among primary consumers[16]. Temperature directly modify the rates of metabolic processes, governing a suit of traits such as population growth rate and food acquisition[17,18], directly affecting the interaction strength between grazers and primary producers through the functional response of food consumption rates[19]. In addition, there is increasing awareness that indirect climate change effects on aquatic food webs also are apparent and important. Particularly the flow of energy, material and organisms across spatially separated ecosystems, commonly referred to as allochthonous inputs or subsidies, can be heavily modified by climate-related factors[20,21]. An interesting aspect of this is that climate change effects on primary producers in terrestrial habitats[22], likely will play a role in determining the production also in aquatic systems.

Here, we test how varying allochthonous input, resulting from spatial variation in vegetation cover, in combination with temperature variation modify key parameters of the dynamics between zooplankton and their phytoplankton resource base. Inference to untangleglobal change effects on trophic interactions include elaborated theoretical simulation models based upon physiological principles, experimental setups, and observational or correlative studies of spatio-temporal changes in natural systems[3,4,11,12,23]. In the current study, we apply all three approaches. We used the climatic variation among arctic lakes as a natural experiment, and analysed trophic interactions with a bioenergetics based framework derived from classical Lotka-Volterra predator-prey models[24]. The lakes were used as a model system to study effect of temperature and land-cover in the catchment (drainage basin) on interactions strength between phytoplankton (using chlorophyll *a* as proxy) and zooplankton populations. We the used a hierarchical Bayesian statistical framework to show how global change affected drivers may alter the interaction strength between phytoplankton and zooplankton populations in ways that are unpredictable using single factor analyses.

The data was collected by field sampling in 20 ponds and lakes (hereafter lakes) in Zackenberg valley and Daneborg, Northeast Greenland (S1 Fig). The lakes were sampled repeatedly throughout the summer season, and seasonal variations in zooplankton and phytoplankton was registered. In addition, the vegetation coverage in the catchment of each lakes was classified visually in subsample quadrants as percentage coverage of 11 landcover classes (rock or gravel, bare soil, mosses, graminoids, *Dryas*, lichens, *Salix*, *Cassiope*, *Saxifraga*, bogs or wetland, and other). This “space-for-time” approach is highly suitable to understand temporal changes caused by global change affected drivers, and has resulted in considerable insight into temporal dynamics in previous studies[25]. The mean temperature in the lakes varied between 10 and 13°C. The variation is likely caused by topological variation within the study area. Each lake was visited four times at regular intervals during the period 2013-07-18 to 2013-08-11 (see S1 Table). Also vegetation coverage and composition have undergone dramatic changes in northern areas during the past few decades[26–28], trends that have been captured both by remote sensing and by on-site observations[28–30].

## Results

**Fig 1:**
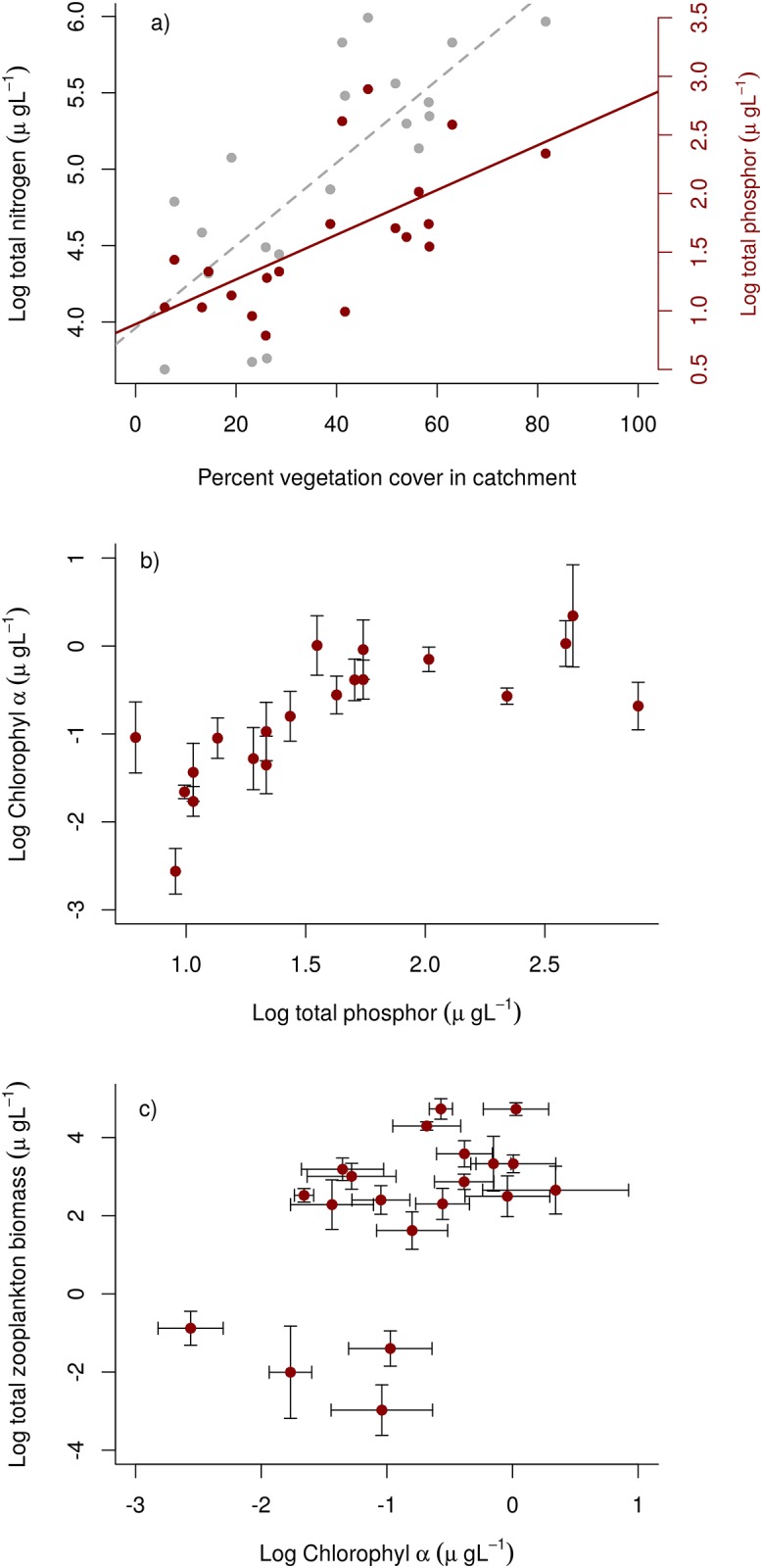
Relationship between catchment vegetation cover, water chemistry, phytoplankton and zooplankton biomass in 20 ponds and lakes in Zackenberg and Daneborg during July and August. **a)** The nutrient content in the ponds increased with vegetation cover. Grey dots and line indicates log(total nitrogen) μgL^-1^, and dark red dots and line indicates log(total phosphorous) μgL^-1^. **b)** Phytoplankton biomass, measured as log(chlorophyll *a*) μgL^-1^, increased with log(total phosporous) μgL^-1^. Not shown in the panel, the relationship bewteen log(chloroplhyll *a*) μgL^-1^ and log(total nitrongen) μgL^-1^ was also significant (*p* = 0.0001). Bars indicate s.e.m. **c)** Zooplankton biomass, measured as log(total zooplankton biomass) μgL^-1^ increased with log (total phytoplankton) μgL^-1^ biomass. Bars indicate s.e.m. Nutrients and biomasses are log-transformed.

The vegetation cover of lake catchments varied between 0 and 100%. The main vegetation coverage consisted of mosses (21%), and vascular plants of the genera *Cassiope* (27%), *Dryas* (15%) and *Salix* (13%). In order to assess the relationship between vegetation coverage and water chemistry variables, we measured total phosphorous and nitrogen in water samples. Both the concentration of total phosphorous (totP, range; 2.2–18.0 μg l^-1^) and total nitrogen (totN, range 40–400 μg l^-1^) varied about one order of magnitude between study lakes. Further, there was a strong relationship between totP- and totN concentration in the lakes, and vegetation coverage of the catchments (Nitrogen: *F* = 25.42, *d*.*f*. = 1,18, *p*<0.001, *r*^2^ = 0.58, linear regression model; phosphorous: *F* = 13.54, *d*.*f*. = 1,18, *p* = 0.0017, *r*^2^ = 0.42, linear regression model: Fig 1a). The relationship between catchment vegetation coverage and total organic carbon (TOC, S2 Fig) was similar to that of totN and totP (*F* = 21.22, *d*.*f*. = 1,18, *p*<0.001, *r*^2^ = 0.54, linear regression model: not shown in figure). We also measured Chlorophyll *a* concentration in water samples as a proxy for primary production. Chlorophyll *a* concentration varied more than two orders of magnitude among lakes (range 0.03–3.14 μg l^-1^), and there was a close correspondence between chlorophyll *a* concentration and P (*χ*^*2*^ = 14.049, *d*.*f*. = 1,18, *p* = 0.0002, linear mixed effects model with lakeID fitted as random intercept term: Fig 1b), and also between chlorophyll *a* concentration and N (*χ*^*2*^ = 14.957, *d*.*f*. = 1,18, *p* = 0.0001, linear mixed effects model with lakeID fitted as random intercept term: Not shown in figure). Finally, zooplankton biomass and chlorophyll a concentration among lakes was positively related (*F* = 6.91, *d*.*f*. = 1, 18, *p* = 0.017, linear regression model through the mean values for each lake: Fig 1c).

**Fig 2:**
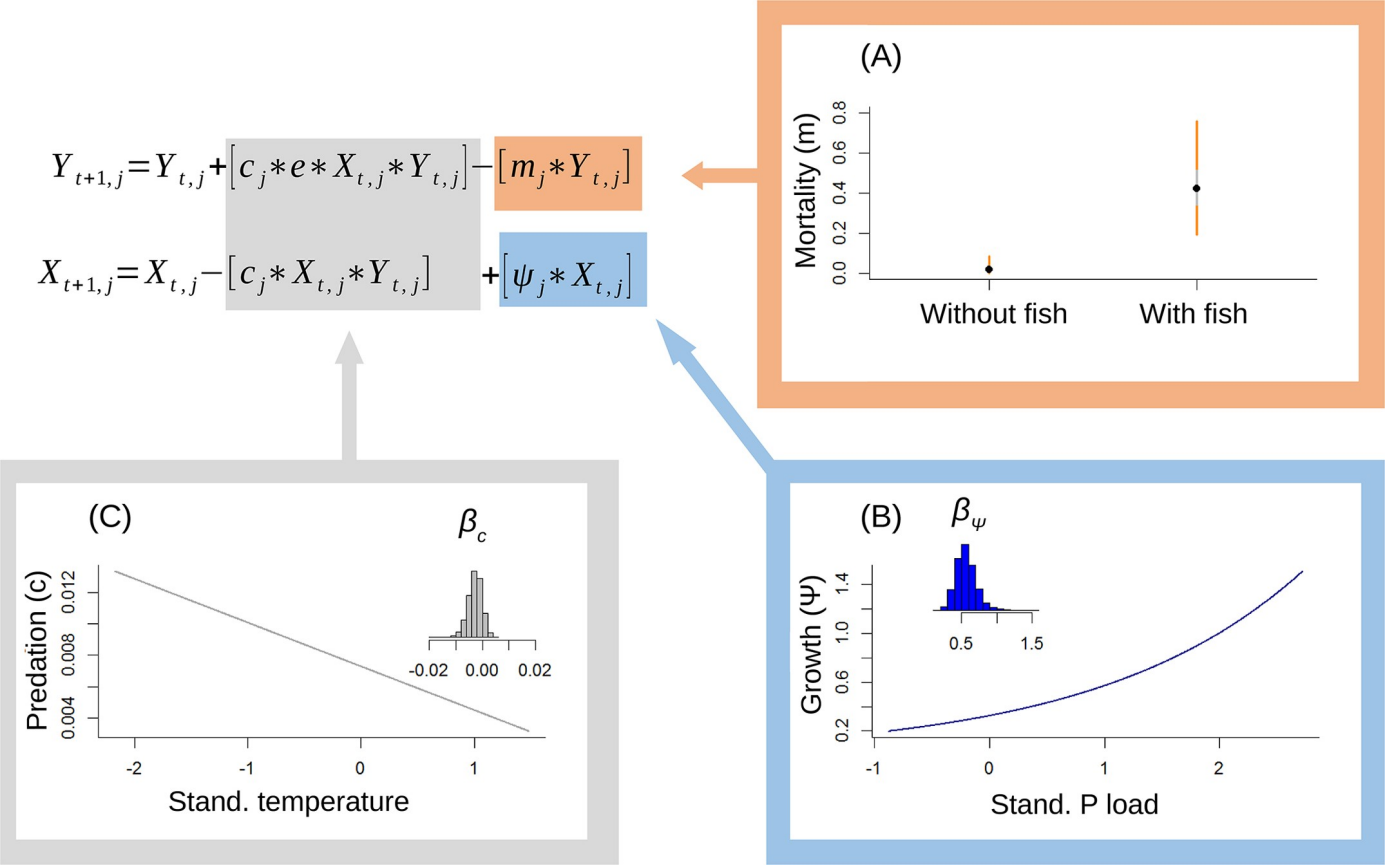
Estimated effects of food web structure, allochthonous material and water temperature on model parameters in the hierarchical state-space predator-prey model. **a)** Estimated relationship between zooplankton mortality rates and presence or absence of fish. **b)** Estimated relationship between phytoplankton growth rate and standardized P load (see main text for original scale of measurement). Inset histogram shows the posterior probability distribution for model parameter *β*_*ψ*_. **c)** Estimated relationship between predation rate of zooplankton on phytoplankton (*c*) and standardized water temperature (see main text for how temperature was measured). Inset histogram shows the posterior probability distribution for model parameter *β*_*c*_. Explanation of equation and parameters is given in Eqs 1 and 2.

The correlative results above suggests that there is a close relationship between allochthonous input, water chemistry and the biological dynamics in arctic lakes. To further examine the dynamic properties of the system, we fitted hierarchical state-space predator-prey models[24,31] to estimate the impact of allochthonous material and temperature on the dynamic interaction between phytoplankton and zooplankton (Eqs 1 and 2 in Material and methods section). There was a clear effect of nutrient level and food chain length (i.e. presence or absence of fish that feed on zooplankton) on the parameters in the predator-prey model. In general, the estimated mortality rate of the zooplankton population (Fig 2a) was more than one order of magnitude lower in lakes without fish (mean of the posterior distribution: 0.03, 95% C.I: 0.001–0.087) compared to lakes with fish (mean of the posterior distribution: 0.44, 95% C.I: 0.18–0.78). It follows from basic predator-prey theory that this will result in a lower predator zero-isocline (i.e. lower predicted equilibrium density and lower grazing rates of zooplankton on phytoplankton) in lakes with fish compared to lakes without fish (Eqs 1 and 2). Further, the maximum growth rate of the phytoplankton was positively related to phosphorous load (Fig 2b), and the credibility interval of the estimated coefficient parameter *β*_*ψ*_ did not overlap zero (mean of the posterior distribution: 0.559, 95% C.I: 0.321–0.864). Finally, there was a clear indication for a negative effect of temperature on predation rate *c* (Fig 2c). The mean of the posterior distribution for the slope parameter *β*_*c*_ was estimated at -0.003 (95% C.I: -0.008–0.002), with 86% of the posterior probability distribution being below zero.

## Discussion

Our study demonstrates how environmental differences similar to changes caused by global change may simultaneously modify trophic interactions and productivity of different tropic levels, either through direct effects from temperature change, or through indirect effects through altered allocthonous import from adjacent ecosystems. Both these effects point to an increase in biomass of primary producers and less potential for top-down control of the system as temperature increases and primary production increases due to increased allocthonous import of nutrients. In addition, presence of apex predators in the system (here predatory fish) resulted in elevated zooplankton mortality, which in turn will result in lower zooplankton growth rate, lower predator zero isocline [24] and thus lower zooplankton equilibrium density. Although the system investigated here is not expected to be in equilibrium in the short arctic summer, general predictions from predator-prey theory then predicts lower prey (i.e. phytoplankton) density with increased predator mortality. This reemphasizes the modification of environmental drivers imposed by altered biodiversity and food chain length. In isolation, the mechanisms demonstrated in this study are previously well documented, including temperature effects on functional responses[19] and trophic interactions in aquatic systems[4,32,33], fish predation on zooplankton communities[34], and catchment vegetation effects on aquatic nutrient load[35] and nutrient mineralisation[36]. However, our integrative approach demonstrate this effects simultaneously operating in the same system, and offers some new insight into the interplay between drivers and underline the value going beyond correlative studies when assessing effects of climate change on ecosystem functioning [13].

Subsidies across the terrestrial-aquatic interface play an important role in governing the input of nutrients and energy to aquatic habitats[37]. Thus, the relative effect of top-down versus bottom-control on system productivity not only depend upon changes in the focal aquatic system, but also in adjacent terrestrial systems[38,39]. The spatial flows of nutrients and organic matter across ecosystem boundaries are therefore likely to play a major role in the responses of ecosystems to climate change[35,40,41]. In addition to nutrients, an increase in terrestrial vegetation cover also cause an increase in organic carbon. Organic carbon has a strong impact on light attenuation and increased carbon load decreases light penetration reducing the euphotic zone and thereby the volume of the lake where primary production can occur[42]. However, allochthonous carbon can also be utilized directly as a food source for microbes and certain algae or mediate the transport of nutrients[43], or lead to anoxic conditions decreasing primary consumption due to the reduction of oxygenated habitat[44]. Our study was not designed to differentiate further among effects of different allochthonous subsidies, which all were strongly intercorrelated (see Fig 1). Also, note that we only included small shallow ponds and lakes in our study. The effect of increased TOC on production is likely to be highly dependent upon the maximum depth and the depth profile of the waterbody[45]. Therefore, although presumably not relevant for the specific lakes in this study, the effect of increasing allochthonous carbon load to freshwaters are likely to be important for many arctic and boreal lakes and acting as a contrasting force to increased nutrient load.

We believe that our integrative approach gives important new insights into the anticipated dynamics of arctic lakes under future climate change scenarios. Previous studies have indicated that the functional response (at least of heterothermic animals) is a humped shaped function of temperature[19]. Contrary to many other studies[46,47,48], we find a negative effect of increasing temperature on grazing rates, and hence top-down control. One possible explanation for this observation is that our study was conducted during mid-summer and that temperatures are in the higher range experienced for these high arctic ecosystem. This corresponds to findings from laboratory studies[49], observing a decrease in *Daphnia* grazing effectiveness at the upper end of the species temperature range and subsequent reduction in top-down control of the phytoplankton community. Climate changes does not only manifest as temperature effects acting through the physiology of individual organisms within the focal system. For aquatic ecosystems, increased allochthonous export of nutrients and carbon from adjacent terrestrial ecosystems affect primary production, as well as biotic interactions, community composition and food chain structure[50,51]. The links between climate and allochthonous export are complex and depend upon a range of factors, including trophic interactions between plants and herbivores in the terrestrial environment. This emphasised that global change impacts on trophic interactions are difficult to predict and forecast. For example, food chain length strongly depends upon the composition of the biotic community, and is expected to change dynamically following climate driven range expansions[6]. Our results suggest that global warming in arctic lakes may result in a weakening of top down control trough increased phytoplankton growth rates and decreased grazing from zooplankton. This might suggest a subsequent greening of arctic lakes parallel to the previously documented greening of the arctic tundra.

## Materials and methods

### Field samples and laboratory analyses

Sampling permission where provided by the Government of Greenland, Ministry of Domestic Affairs, Nature and Environment. Permit number: C-13-4(13).

Zooplankton was sampled by filtering 20 l of water through a 90 μm plankton net. The water samples where from the deepest point of the lake, or as far out as accessible with waders (ca. <1.5 m depth). Three replicated samples per lake were collected at each sampling day. Zooplankton was stored in 70% ethanol. All organisms in a sample were counted, with exception of some samples where naupli larvae was counted using subsamples. In this case, a known fraction was examined until at least 200 organisms were counted. *Cyclops* spp. were identified to genus level, whereas the other main zooplankton taxa (*Macrothrix hirsuticornis*, *Daphnia pulex*, *Chydorus sphaericus*) were determined to species level. Rare species or singletons (*Harpacticoida* spp., *Alona rectangular*, *Bosmina longispina*) that compromised less than 1% of samples in each lake were not identified further and not included in the analyses. Biomass of zooplankton was determined by multiplying individual biomass estimated from a sub-sample of individuals in one of the replicates at each lake at each sampling occasion (25 individuals or as many as in sample if less) with the total count. Each individual from the sub-sample was length measured under a dissection microscope, and dry mass (μg) was determined using length-weight regression following[52], and in case of naupli larva[53]. Information on presence or absence of fish (Arctic char, *Salvelinus alpinus* L.) was based on previous gillnet test fishing in the lakes[54,55].

Water samples for chlorophyll *a* and chemical analysis were taken as surface samples over the deepest point in the lake, or as far out as accessible with waders (where the water was ca. <1.5 m deep). Water samples for chemical parameters were frozen at -18°C at the day of sampling and later analyzed for total organic carbon (TOC), total nitrogen (totN) and total phosphorous (totP) according to standard procedures in an accredited (NS EN ISO 17025) water laboratory (Analysesenteret, https://www.trondheim.kommune.no/analysesenteret/). Chlorophyll *a* were extracted from filtered water samples according to standard methods (ISO 10 260) at the Department of Biology, Norwegian University of Science and Technology. Water samples used for chlorophyll *a* measures (1–2 liters) were kept dark and cool and filtered (Whatmann GF/C) using a suction pump, on the day of collection, following the protocol in[56]. Filters were then dried and packed in aluminium foil, wrapped in plastic and immediately frozen at -18°C at the field lab. The samples were kept frozen until returning to Norway and there stored at -70°C until analysed. Chlorophyll *a* concentrations were measured by photometrical absorbance at 665 and 750 nm after solving filters in ethanol[57]. One of the lakes had extreme deviating levels of nutrients (lakeID 2). This pond was observed frequently visited by geese (*Anatidae* spp.) and large amounts of faeces were recorded on the substrate of the pond and in the surrounding terrain. Data from this pond were hence removed from the statistical analyses.

Vegetation coverage of lake catchment areas were determined by walking transects in four directions (north, east, south and west) to the end of the catchment or 200 meters (whatever came first). A minimum of four plots per transect (2 square meters) were set at regular intervals and percentage coverage of 11 classes (rock or gravel, bare soil, mosses, graminoids, *Dryas*, lichens, *Salix*, *Cassiope*, *Saxifraga*, bogs or wetland, and other) were determined by visual inspection. Mean coverage of vegetation classes throughout all sampling plots belonging to a particular lake were then used as proxy for catchment vegetation cover of that lake.

Temperature were logged in each lake with temperature loggers (HOBO pendant, UA-002; Onset Computer) placed 0.3 meters above the bottom substrate at 1.5 meters depth (or deepest point of the lake, if shallower). Loggers were placed in the lake at the time of first sampling and recollected after the last sampling.

We initially explored the relationships between percentage vegetation coverage in catchment and water chemistry (totP, totN and TOC), chl a and water chemistry (totP nad totN), and chl a and zooplankton biomass both visually (Fig 1) and statistically. The relationship between chlorophyll *a* concentration and total nitrogen and total phosphorous were analysed using linear mixed effect models with chlorophyll *a* as dependent variable and lakeID as random intercept. The other relationships where analysed using ordinary linear least square regression with values averaged over lakes. With regard to the relationship between vegetation coverage and water chemistry, there was only one sample of vegetation coverage during the study period (one season), yielding mixed effect approach invalid and linear mixed effect models. The relationship between chl a and zooplankton involves multiple time series, yielding a complex model. The statistics in the section refereed to here mainly focuses on providing support to the visualization (Fig 1). More elaborated analyses were done using hierarchical state-space predator-prey model (described below). We did not find any effects of fish presence on the relationships explored in the initial analyses.

### Modeling framework and parameter estimation

Using a survey design of repeated samples in 20 different lakes throughout one season we fitted a hierarchical state-space predator-prey model and estimated model parameters using Bayesian inference and MCMC subsampling from the posterior distributions[31]. The basic equations linking phytoplankton density (indexed based on chlorophyll *a* content) and zooplankton (see above for methods to estimate zooplankton density) were

$$Y_{t+1},j=Y_{t,j}+\left[c_{j}*e*X_{t,j}*Y_{t,j}\right]–\left[m_{j}*Y_{t,j}\right]$$ (1)

and

$$X_{t+1,j}=X_{t,j}+\left[\psi_{j}*X_{t,j}\right]–\left[c_{j}*X_{t,j}*Y_{t,j}\right]$$ (2)

where *Y*_*t*,*j*_ is the zooplankton abundance at time *t* in lake *j* and *X*_*t*,*j*_ is phytoplankton abundance at time *t* in lake *j*. The other parameters determining the dynamics of the system, are:

*c*_*j*_: Consumption parameter in lake *j*,
*ψ*_*j*_: Population growth rate of phytoplankton in lake *j*,
*m*_*j*_: Mortality rate of zooplankton population.in lake *j*,
e: Energy efficiency of the zooplankton population.

Because we only had four measurements for each lake, we did not consider time-dependent covariates, but did allow coefficients *c*, *ψ and m* in Eqs 1 and 2 to vary between lakes, depending on their measured values of the covariates. More precisely, we allowed predation rate (*c*) to be dependent on temperature, and plankton population growth (*ψ*) rate to be a function of *P* load (*P* commonly assumed to be the limiting factor in freshwaters). The effect of fish mortality on zooplankton mortality (*m*_*j*_) was modelled by including fish presence/absence as a two-factor variable, allowing *m*_*j*_ to vary between lakes with and without fish. Prior to fitting the models, we standardized the covariates water temperature and phosphorous load (scale of measurement: μg phosphorous L-1) by subtracting the mean and dividing on the standard deviation[58]. The standardized temperature (ST) and phosphorous load (SP) were then included in the hierarchical predator-prey model by fitting linear relationship on the form *c*_*j*_ = *α*_*c*_ + *β*_*c*_*ST* [eg. 3] and log(*ψ*_*j*_) = *α*_*ψ*_ + *β*_*ψ*_*SP* [eg. 4].

Because all ecological time series contain measurement error, we related the field measurements of phytoplankton abundance (μgL^-1^) and zooplankton (μgL^-1^) in lake *j* at time *t* assuming they were sampled from a normal distribution with means equal to *X*_*t*,*j*_ and *Y*_*t*,*j*_ respectively, and variances equal to *σ*_*x*_ and *σ*_*y*_ respectively. Lake-specific time series of field measurements of phytoplankton abundance (μgL^-1^), zooplankton (μgL^-1^) and estimates of *X*_*t*,*j*_ and *Y*_*t*,*j*_ respectively is included in S1 and S2 Figs.

Model parameters were estimated using Bayesian inference[31], running program jags from the free statistical software R[59] using add-on library R2jags[60]. We ran three chains, with a total of 250.000 iterations, an initial burn-in of 100.000 iterations, and a thinning rate equal to 3. Convergence was assessed using R-hat statistics, and visually inspected (S5 Fig) using the function traceplot in add-on library R2jags[60].

## Supporting information

S1 Table Sampling locations with lake characteristics. Given with coordinates (decimal latitude and longitude in WGS84, EPSG:4326), elevation (m.a.s.l), perimeter (meters), Area (square meters), Maximum depth (cm), presence or absence of fish and sampling dates (day.month, 2013) for zooplankton, chlorophyll *a*, and water samples.
(PDF)

S1 Fig Map of study area in Northeast Greenland. Online presentation, including site-specific details is found on https://goo.gl/DnzBLM. Archived at Zenodo: DOI: 10.5281/zenodo.31268.
(PDF)

S2 Fig Vegetation cover and lake total organic carbon. Relationship between percentage vegetation cover in catchment area and log(total organic carbon) μg l^-1^. The relationship is significant (F = 21.22, d.f. = 18,1, p = 0.0002, linear regression model).
(PDF)

S3 Fig Observed and estimated zooplankton biomass. Time series plot of field measurements of zooplankton biomass (red line) and estimated biomass of zooplankton for lake *j* at time *t* (Y_*j*,*t*_) based on the hierarchical state-space predator-prey model (black lines) for the lakes included in the analysis.
(PDF)

S4 Fig Observed and estimated phytoplankton concentration. Time series plot of field measurements of phytoplankton concentration (red line) and estimated phytoplankton biomass for lake *j* at time *t* (X_*j*,*t*_) based on the hierarchical state-space predator-prey model (black lines) for the lakes included in the analysis.
(PDF)

S5 Fig Iterations *vs*. sampled values for model parameters in the MCMC chains. The three different chains are plotted using different colours. Plots were generated using function *traceplot* in add-on library R2jags (Su, Y.-S. and M. Yajima, 2015. "R2jags: Using R to Run 'JAGS'." http://CRAN.R-project.org/package=R2jags).
(PDF)

S1 Text R code for data preparation. (R)

S2 Text R code for JAGS analyses. (R)
