# Supplementary material for: Catchment vegetation and temperature mediating trophic interactions and production in plankton communities
Source: PLoS One. 2017 Apr 17;12(4):e0174904. doi: 10.1371/journal.pone.0174904 (PMC5393547; doi:10.1371/journal.pone.0174904)
Supplement: S1 Table — Given with coordinates (decimal latitude and longitude in WGS84, EPSG:4326), elevation (m.a.s.l), perimeter (meters), Area (square meters), Maximum depth (cm), presence or absence of fish and sampling dates (day.month, 2013) for zooplankton, chlorophyll a, and water samples. (PDF) [file pone.0174904.s001.pdf]

Supplementary information for "Catchment vegetation and temperature determine the strength of trophic interactions between zooplankton and phytoplankton"

Table S1: Sampling locations with lake characteristics, given with coordinates (decimal latitude and longitude in WGS84, EPSG:4326), elevation (m.a.s.l), perimeter (meters), Area (square meters), Maximum depth (cm), presence or absence of fish and sampling dates (day.month, 2013) for zooplankton, chlorophyll *a*, and water samples.

locationID	Latitude	Longitude	Elevation	Perimeter	Area	Maximum depth	Fish	Dates
1	74.3231054	-20.184435	110	1100	69783	>300	yes	18.7,26.7,1.8,7.8
2	74.3067914	-20.199207	46	785	23185	60	no	19.7,25.7,1.8,7.8
3	74.310437	-20.094494	51	1100	67745	>150	yes	20.7,25.7,31.7,6.8
4	74.3134634	-20.113445	53	976	42348	>150	yes	20.7,25.7,31.7,6.8
5	74.3164274	-20.09724	50	457	7910	>150	no	20.7,25.7,31.7,6.8
6	74.3188639	-20.10781	54	773	31459	>150	no	20.7,25.7,31.7,6.8
7	74.3190352	-20.125934	46	1600	100000	>300	yes	20.7,25.7,31.7,6.8
8	74.3584902	-20.163928	181	686	17756	>300	no	21.7,26.7,1.8,7.8
9	74.3604458	-20.169078	185	740	27565	>300	no	21.7,26.7,1.8,7.8
10	74.5086152	-20.628422	93	599	16453	670	yes	23.7,29.7,4.8,10.8
11	74.5067248	-20.628903	106	182	2486	70	no	23.7,29.7,4.8,10.8
12	74.5063182	-20.626967	140	334	6140	170	no	23.7,29.7,4.8,10.8
13	74.5061893	-20.624924	148	618	22462	460	yes	23.7,29.7,4.8,10.8
14	74.5041389	-20.626247	183	303	4175	150	no	23.7,29.7,4.8,10.8
15	74.5027174	-20.628393	58	301	5184	340	no	23.7,29.7,4.8,10.8
16	74.5029974	-20.624329	72	213	2569	50	no	23.7,29.7,4.8,10.8
17	74.5039461	-20.607943	71	352	7009	150	no	23.7,29.7,4.8,10.8
18	74.5044679	-20.603973	74	613	17041	270	no	23.7,29.7,4.8,10.8
19	74.5005618	-20.605042	101	477	16940	610	yes	23.7,29.7,4.8,10.8
20	74.4986804	-20.600497	109	642	17342	180	no	23.7,29.7,4.8,10.8
21	74.477994	-20.553879	38	221	2671	>150	no	24.7,30.7,5.8,11.8
