# Supplementary figures and images for: Catchment vegetation and temperature mediating trophic interactions and production in plankton communities
Source: PLoS One. 2017 Apr 17;12(4):e0174904. doi: 10.1371/journal.pone.0174904 (PMC5393547; doi:10.1371/journal.pone.0174904)

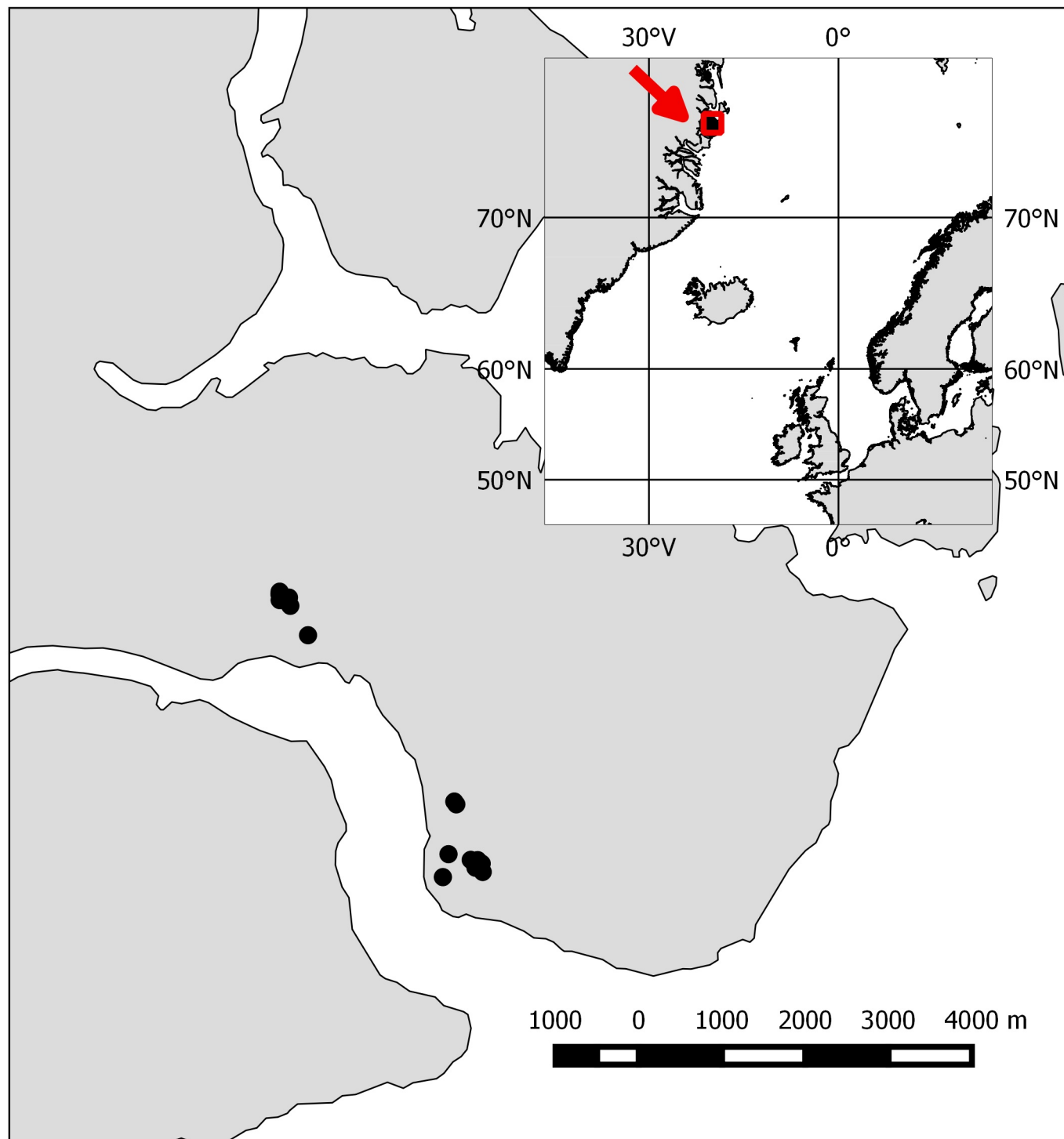

Supplement: S1 Fig — Online presentation, including site-specific details is found on https://goo.gl/DnzBLM. Archived at Zenodo: DOI: 10.5281/zenodo.31268. (PDF) [file pone.0174904.s002.pdf]
