# Supplementary material for: Catchment vegetation and temperature mediating trophic interactions and production in plankton communities
Source: PLoS One. 2017 Apr 17;12(4):e0174904. doi: 10.1371/journal.pone.0174904 (PMC5393547; doi:10.1371/journal.pone.0174904)
Supplement: S2 Fig — Relationship between percentage vegetation cover in catchment area and log(total organic carbon) μg l-1. The relationship is significant (F = 21.22, d.f. = 18,1, p = 0.0002, linear regression model). (PDF) [file pone.0174904.s003.pdf]

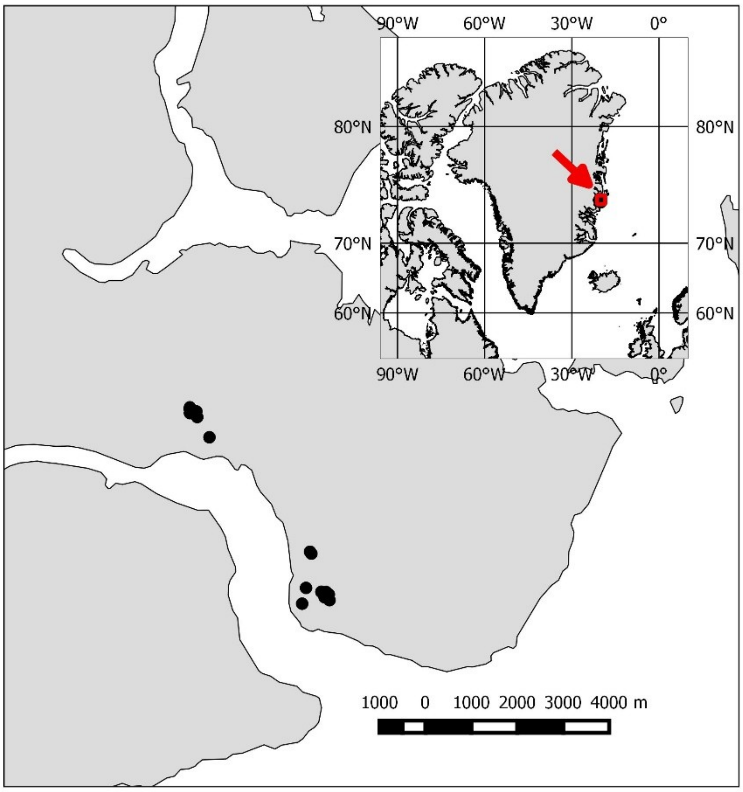


**Fig S1**: Map of study area in Northeast Greenland with black dots indicating individual study sites sampled for phytoplankton, zooplankton, water chemistry, temperature and catchment vegetation cover during the summer 2013. An online presentation, including site-specific details is found on https://goo.gl/DnzBLM (DOI: 10.5281/zenodo.31268). Figure made using QGIS version 2.12.2-Lyon; URL http://qgis.org. © OpenStreetMap contributors, URL http://www.openstreetmap.org/copyright/en.
