# Supplementary material for: Catchment vegetation and temperature mediating trophic interactions and production in plankton communities
Source: PLoS One. 2017 Apr 17;12(4):e0174904. doi: 10.1371/journal.pone.0174904 (PMC5393547; doi:10.1371/journal.pone.0174904)
Supplement: S3 Fig — Time series plot of field measurements of zooplankton biomass (red line) and estimated biomass of zooplankton for lake j at time t (Yj,t) based on the hierarchical state-space predator-prey model (black lines) for the lakes included in the analysis. (PDF) [file pone.0174904.s004.pdf]

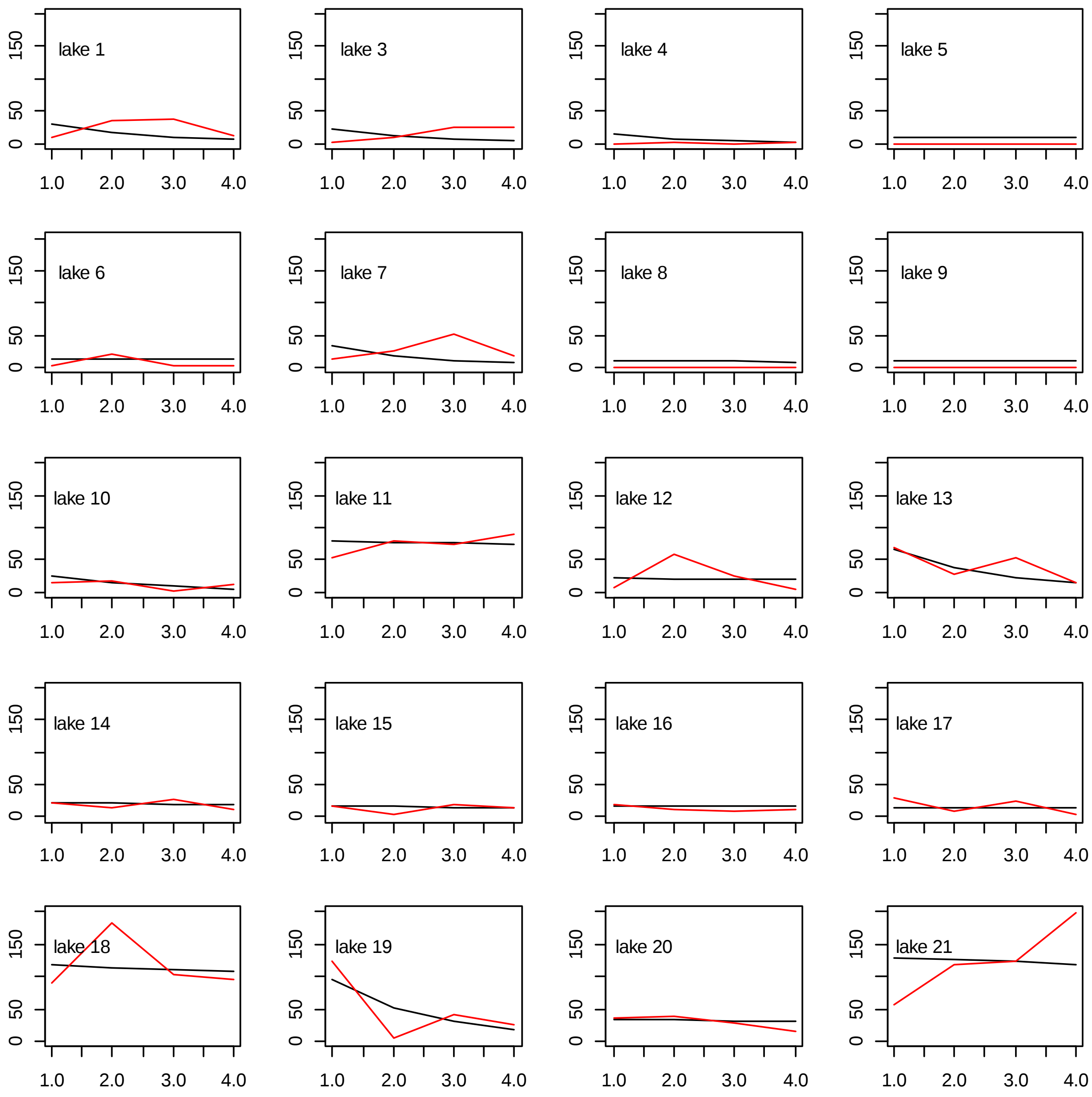

Fig S3: Time series plot of field measurements of zooplankton biomass (red line) and estimated biomass of zooplankton for lake *j* at time *t* ($Y_{j,t}$) based on the hierarchical state-space predator-prey model (black lines) for the lakes included in the analysis.
