# Supplementary material for: Catchment vegetation and temperature mediating trophic interactions and production in plankton communities
Source: PLoS One. 2017 Apr 17;12(4):e0174904. doi: 10.1371/journal.pone.0174904 (PMC5393547; doi:10.1371/journal.pone.0174904)
Supplement: S4 Fig — Time series plot of field measurements of phytoplankton concentration (red line) and estimated phytoplankton biomass for lake j at time t (Xj,t) based on the hierarchical state-space predator-prey model (black lines) for the lakes included in the analysis. (PDF) [file pone.0174904.s005.pdf]

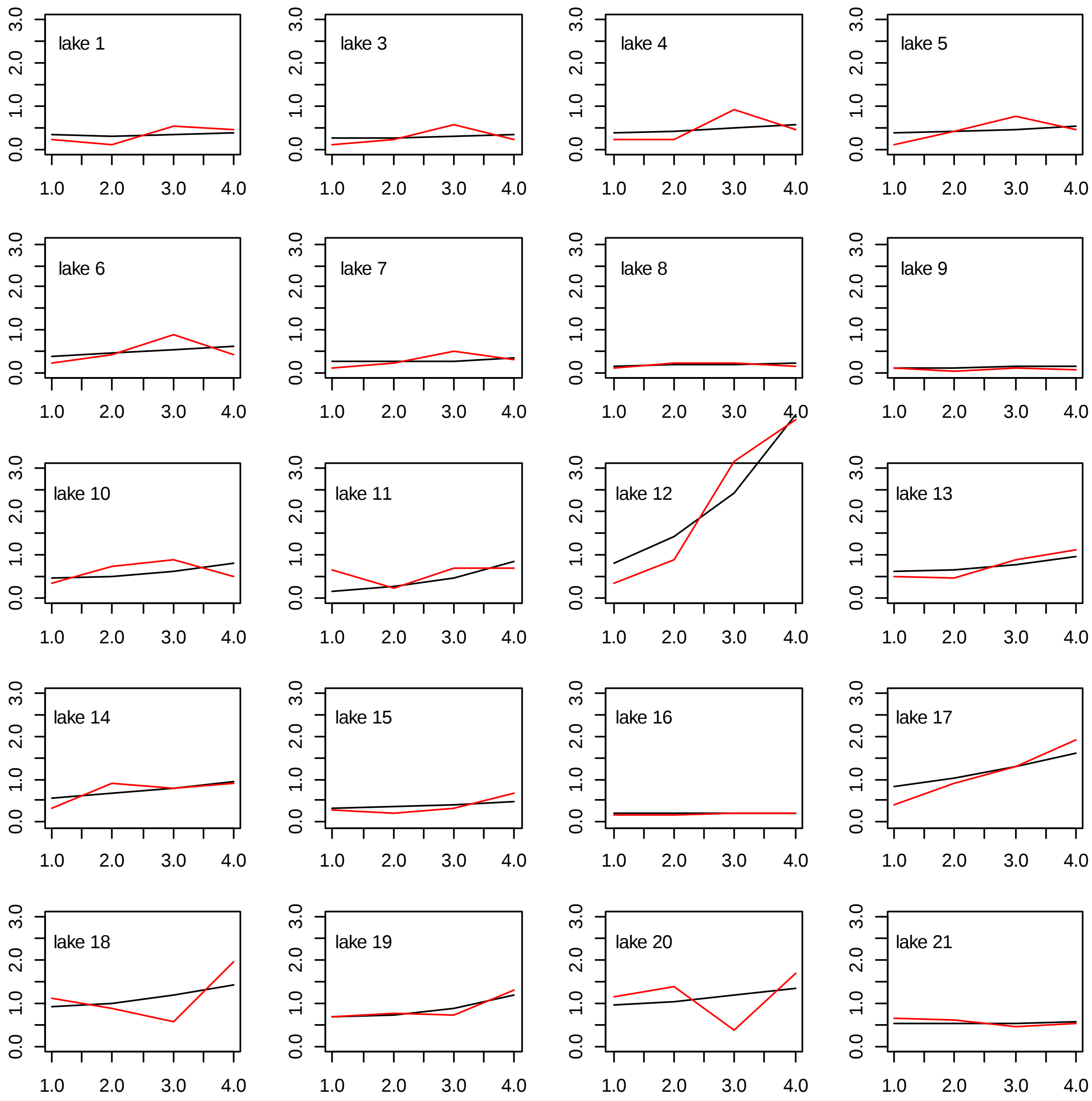


**Fig. S4:** Time series plot of field measurements of phytoplankton concentration (red line) and estimated phytoplankton biomass for lake *j* at time *t* ($X_{j,t}$) based on the hierarchical state-space predator-prey model (black lines) for the lakes included in the analysis.
