# Supplementary material for: Catchment vegetation and temperature mediating trophic interactions and production in plankton communities
Source: PLoS One. 2017 Apr 17;12(4):e0174904. doi: 10.1371/journal.pone.0174904 (PMC5393547; doi:10.1371/journal.pone.0174904)
Supplement: S5 Fig — The three different chains are plotted using different colours. Plots were generated using function traceplot in add-on library R2jags (Su, Y.-S. and M. Yajima, 2015. "R2jags: Using R to Run 'JAGS'." http://CRAN.R-project.org/package=R2jags). (PDF) [file pone.0174904.s006.pdf]

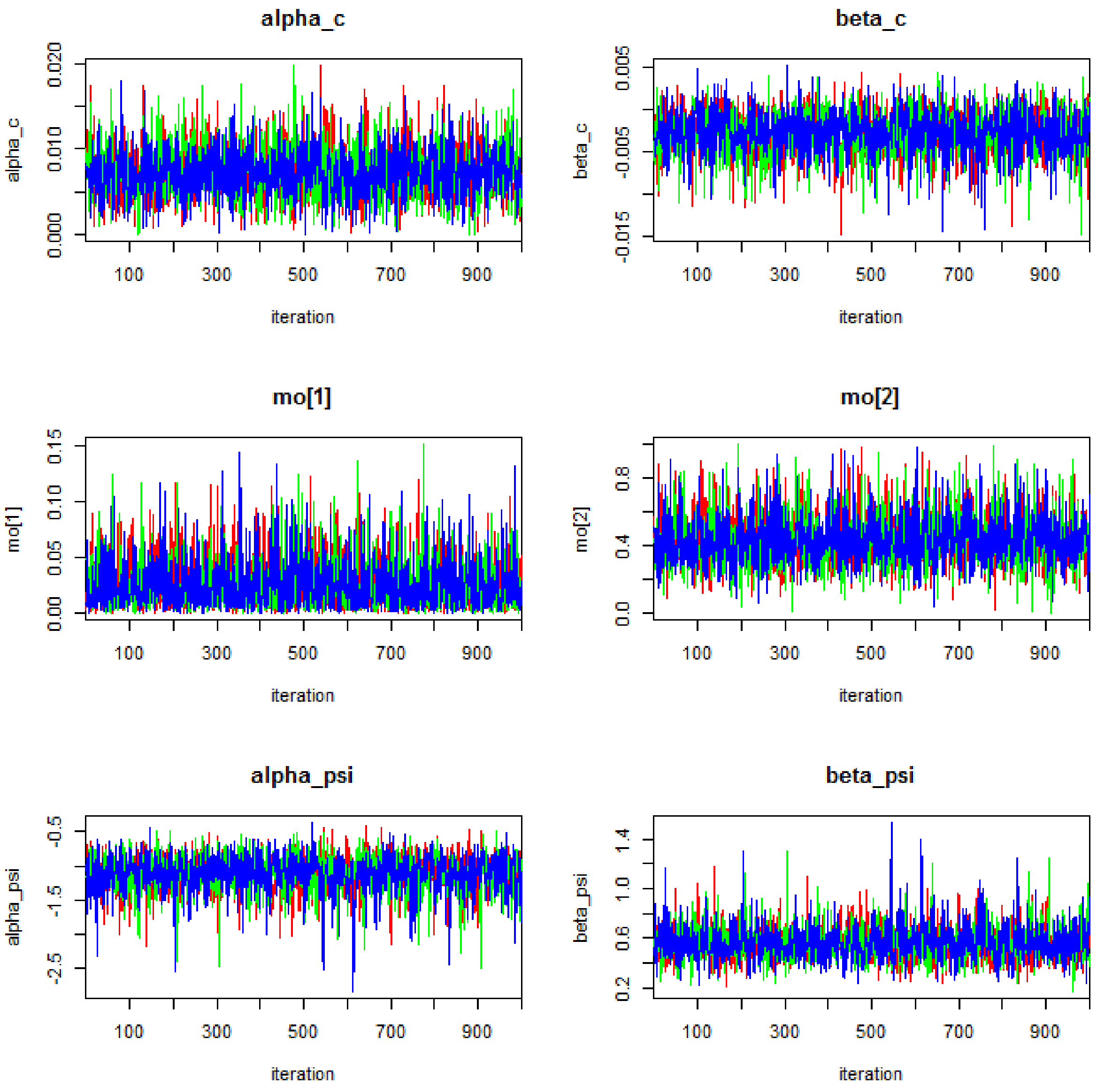

Fig. S5. Plot of iterations *vs.* sampled values for model parameters in the MCMC chains. The three different chains are plotted using different colours. Plots were generated using function *traceplot* in add-on library R2jags (Su, Y.-S. and M. Yajima, 2015. "R2jags: Using R to Run 'JAGS'." http://CRAN.R-project.org/package=R2jags).
